# Gut microbiota: a new player in regulating immune- and chemo-therapy efficacy

**DOI:** 10.20517/cdr.2020.04

**Published:** 2020-03-21

**Authors:** Simone Anfossi, George A. Calin

**Affiliations:** 1 Department of Experimental Therapeutics, The University of Texas MD Anderson Cancer Center, Houston, TX 77054, USA.; 2 Center for RNA Interference and Non-coding RNAs, The University of Texas MD Anderson Cancer Center, Houston, TX 77054, USA.

**Keywords:** Gut microbiota, resistance, chemotherapy, immunotherapy, immune checkpoint inhibitor, extracellular vesicles, miRNAs, cell-to-cell communication

## Abstract

Development of drug resistance represents the major cause of cancer therapy failure, determines disease progression and results in poor prognosis for cancer patients. Different mechanisms are responsible for drug resistance. Intrinsic genetic modifications of cancer cells induce the alteration of expression of gene controlling specific pathways that regulate drug resistance: drug transport and metabolism; alteration of drug targets; DNA damage repair; and deregulation of apoptosis, autophagy, and pro-survival signaling. On the other hand, a complex signaling network among the entire cell component characterizes tumor microenvironment and regulates the pathways involved in the development of drug resistance. Gut microbiota represents a new player in the regulation of a patient’s response to cancer therapies, including chemotherapy and immunotherapy. In particular, commensal bacteria can regulate the efficacy of immune checkpoint inhibitor therapy by modulating the activation of immune responses to cancer. Commensal bacteria can also regulate the efficacy of chemotherapeutic drugs, such as oxaliplatin, gemcitabine, and cyclophosphamide. Recently, it has been shown that such bacteria can produce extracellular vesicles (EVs) that can mediate intercellular communication with human host cells. Indeed, bacterial EVs carry RNA molecules with gene expression regulatory ability that can be delivered to recipient cells of the host and potentially regulate the expression of genes involved in controlling the resistance to cancer therapy. On the other hand, host cells can also deliver human EVs to commensal bacteria and similarly, regulate gene expression. EV-mediated intercellular communication between commensal bacteria and host cells may thus represent a novel research area into potential mechanisms regulating the efficacy of cancer therapy.

## INTRODUCTION

The development of resistance to cancer therapies represents a major challenge in the treatment of cancer patients and the main cause of poor prognosis. Despite initial treatment response, cancer cells can eventually develop resistance, which can result from the acquisition of intrinsic characteristics of cancer cells, and the action of extrinsic factors. Such intrinsic cancer cell characteristics include genetic alterations (mutations, amplifications, deletions, translocations) and epigenetic modifications (methylation, acetylation) that can determine the aberrant expression of genes controlling drug metabolism (inactivation, efflux, target modification), cancer cell survival (inhibition of cell death, pro-survival signaling), or the acquisition of cancer stem cell phenotypes^[1–9]^. Extrinsic factors include the activation of signaling pathways in the tumor microenvironment (TME) that drive the acquisition of drug resistance in cancer cells. For instance tumor-associated immune cells, fibroblasts, and cancer cells can all secrete soluble signaling factors (e.g., cytokines and growth factors) that can activate cancer cell signaling pathways in autocrine or paracrine fashion to induce drug resistance (mTOR, NF-κB, AKT, STAT3)^[10–12]^. Furthermore, the combination of intrinsic and extrinsic factors plays a role in the regulation of tumor cell response to immunotherapy^[13–17]^. More recently, a new and important player is emerging in the regulation of the development of drug resistance: the gut microbiota.

Studies investigating the role of gut microbiota in regulating treatment response to chemotherapy and immunotherapy have been gaining interest. In particular, growing evidence highlights the importance of the interactions between commensal bacteria with both cancer and immune cells in modulating the efficacy of cancer treatment^[18–21]^ [Table 1]. The gut microbiota consists of a multispecies microbial community composed of bacteria, yeast, fungi protozoa, archea, and viruses that establishes symbiosis with the host organism. Our intestinal tract can host up to 100 trillion microbes with the number of expressed genes estimated to outnumber that of the host human’s genes by at least two orders of magnitude^[22,23]^. Recently, the bacterial to human cell ratio has been revised with an updated estimate of ≈ 1:1^[24]^. These studies provide evidence that gut microbiota may influence the physiology of the human host. The microbiota is an important source of metabolites (e.g., vitamins, organic acids, lipids, polyphenols, amino acids, and short chain fatty acids) that exert important functions on the regulation of intestinal epithelial and immune cell metabolism^[25,26]^. These metabolites are involved in the regulation of several biological functions, including modulation of chemotherapy and immunotherapy efficacy. Besides metabolites, bacterial components [lipopolysaccharides (LPS), bacterial cell wall constituents, DNA, and RNA] can also activate innate immune cells^[27,28]^ and modulate the efficacy of cancer treatment (immunotherapy and chemotherapy).

**Figure 1.**
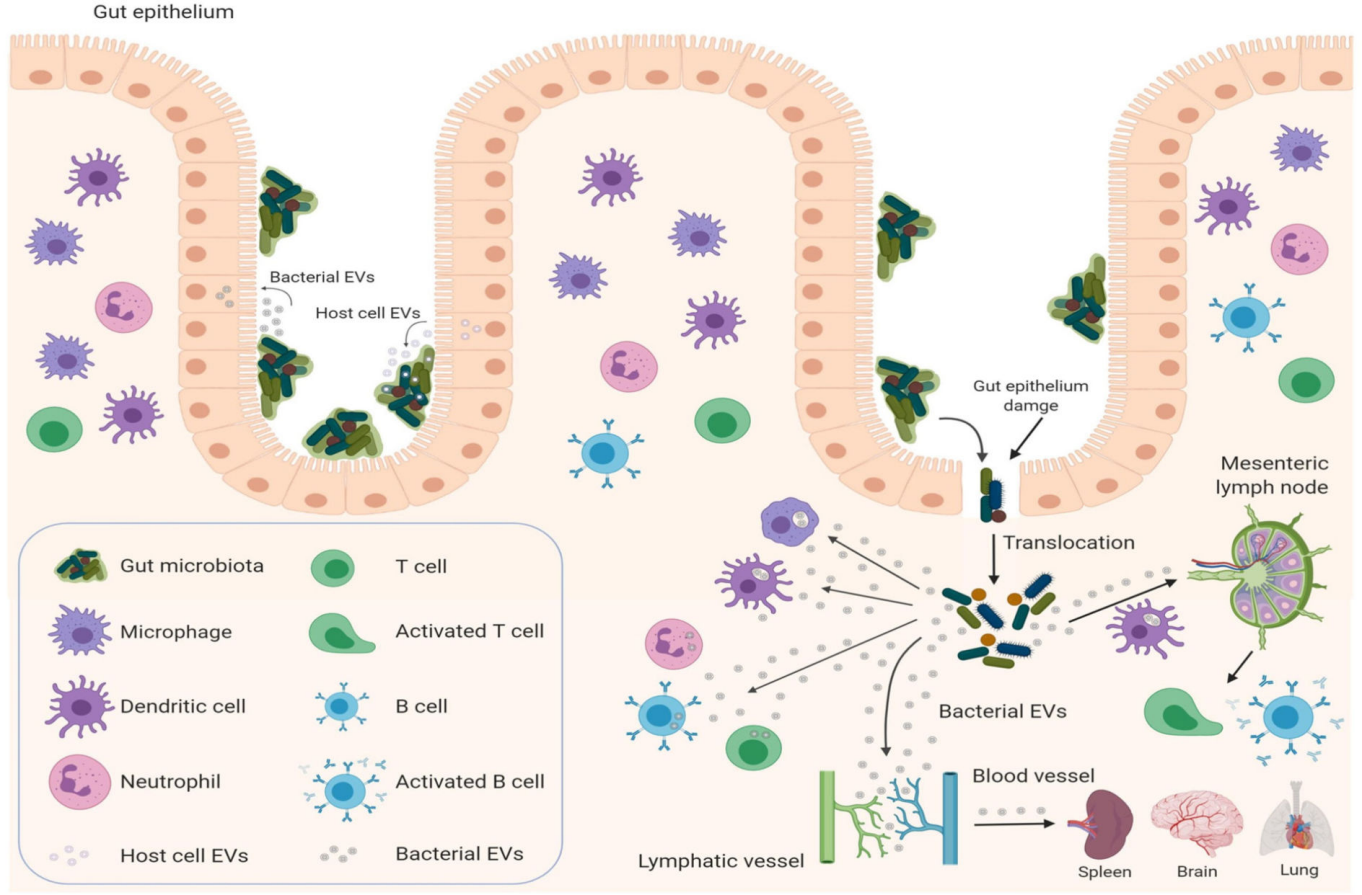
Gut microbiota and host cells: EV-mediated crosstalk. Bacterial EVs can deliver functional and active RNA molecules to host cells, such as gut epithelial cells, immune cells, and potentially cancer cells, and regulate their biological functions by affecting gene expression in recipient cells. Microbe-associated molecular patterns carried by bacterial EVs can activate the immune system locally (mesenteric lymph nodes) or systemically by reaching the circulatory system (spleen, peripheral lymph nodes) to modulate cancer immune responses. Host cells, such as intestinal epithelial cell can affect the gut microbiota by secreting EV-encapsulated miRNAs, which can regulate intestinal bacterial functions and composition by targeting microbial gene expression and potentially modulate the responses to cancer treatment. (Figure is created with BioRender.com). EVs: extracellular vesicles

Extracellular vesicles (EVs) represent an additional level of complexity in the regulation of cancer treatment response. EVs are natural nanoparticles delimited by cellular membranous components that carry lipids, carbohydrates, signaling molecules, metabolites, proteins, DNA, RNA, and mediate cell-to-cell communication by delivering their cargo to recipient cells^[29]^. They are produced and secreted by virtually all cell types in all life kingdoms, including eukaryotic cells^[30]^, prokaryotic cells^[31]^, fungi^[32]^, and archaea^[33]^. Growing evidence suggests that EVs may represent a universal mechanism of cell-to-cell communication in the same (intrakingdom) or different kingdoms (interkingdom) and regulate gene expression in recipient cells^[34]^. To illustrate, bacterial EVs have the ability to mediate horizontal transfer of nucleic acid molecules (intrakingdom communication) and confer antibiotic resistance to recipient cells^[35–39]^. Recently, the interkingdom interactions between bacteria and their human host have aroused particular interest, as bacterial EVs and their RNA cargos have the potential to regulate specific genes in the human host cells^[34]^. EVs produced by the commensal bacteria can cross the intestinal epithelium, where they can regulate the underlying immune cells. Furthermore, bacterial EVs carrying nucleic acids (DNA and RNA) can be secreted into the circulation^[40–43]^, detected in human bodily fluids^[44,45]^, and possibly be delivered to various tissues [Figure 1] after crossing intestinal epithelium^[46]^. Therefore, bacteria can potentially regulate the biological functions of host cells through the delivery of their EVs^[34,46–49]^. Indeed, bacterial EVs can be taken up by eukaryotic host cells and modulate the gene expression of recipient cells^[34,45,50–53]^. How can this be achieved? Bacterial EVs are enriched with short RNAs (sRNAs) ranging around 50–250 nucleotides^[41]^. These sRNAs have regulatory functions similar to miRNAs in eukaryotic cells, as they act by base-pairing with target mRNAs. However, different from eukaryotic miRNAs, sRNAs can either positively or negatively regulate the stability and translation of the target mRNA^[54]^. Recently, sRNAs comparable in size to miRNAs (15–28 nucleotides) named miRNA-sized sRNAs (msRNAs), have been identified in bacteria and may have a similar regulatory function to that of eukaryotic miRNAs^[55–57]^. Therefore, bacterial EVs have the potential ability to regulate specific genes in recipient cells. An intriguing speculation might consider that bacterial EVs could perhaps, similarly modulate the response to cancer therapy of human cells in the TME^[58]^.

Herein, we review the role of gut bacteria in the regulation of cancer treatment response to chemotherapy and immunotherapy, and the different mechanisms of inter-cellular communication between bacterial and host cells and their potential effect on treatment resistance.

## GUT MICROBIOTA REGULATES EFFECTOR FUNCTIONS AND RESPONSES OF IMMUNE CELL TO CHECKPOINT INHIBITORS

The importance of microbes in cancer treatment was first discovered back in 1890, when heat-inactivated *Streptococci* were injected intratumorally^[59]^. Several decades later, it was found that intravesical administration of attenuated *Mycobacterium bovis* (strain *Bacille Calmette-Guérin*, BCG) was able to reduce tumor progression in muscle invasive bladder cancer^[60]^. More recently, it has been discovered that oral administration of *Lactobacillus casei* was able to decrease the recurrence of superficial bladder cancer^[61]^. These evidences highlight the potential role of bacteria in regulating cancer therapy.

Evidence of the functional role of gut microbiota in regulating the efficacy of immunotherapy was first provided by Iida *et al.*^[62]^. Antibiotic treatment (vancomycin, imipenem, neomycin) that disrupted the microbiota or germ-free condition before tumor inoculation impaired the efficacy of CpG-oligodeoxynucleotides and anti-IL-10R treatment in retarding tumor growth and prolonging survival. Gene expression analysis of tumors from mice pre-treated with antibiotics revealed downregulation of genes associated with inflammation, phagocytosis, antigen presentation, and adaptive immune responses, and upregulation of genes related to tissue development, cancer, and metabolism. In particular, antibiotic treatment reduced the infiltration of myeloid-derived cells (monocytes, macrophages, and dendritic cells) and their ability to produce pro-inflammatory cytokines (TNF-α, IL-1α, IL-1β, IL-12, and CXCL10), but not the expression of anti-inflammatory cytokines (IL-10, IL-1RA). The activation of tumor-associated myeloid-derived cells through LPS-mediated activation of TLR4 by commensal microbiota was necessary to prime these cells for the TLR9-dependent response to GpG-oligodeoxynucleotide therapy and achieve optimal response to cancer therapy.

Following this discovery, several studies have shown the key role of gut microbiota in regulating the efficacy of immune checkpoint inhibitor (ICI) therapy for cancer treatment. Vétizou *et al.*^[63]^ found that gut microbiota composition could affect the response to anticancer immunotherapy by CTLA-4 blockade. In an animal model using three different cancer types (sarcoma, melanoma, and colon cancer), the administration of anti-CTLA-4 was able to control tumor growth in mice housed in specific pathogen-free conditions and in the presence of specific *Bacteroides*, but failed to inhibit tumor growth in germ-free mice and mice previously treated with antibiotics. These findings suggest that gut microbiota could potentially regulate the anticancer effect of anti-CTLA-4 therapy. Specific *Bacteroides* species (*B*. *thetaiotaomicron* and *B*. *fragilis*) were able to restore the anticancer response to anti-CTLA-4 treatment when used to recolonize germ-free mice and mice treated with antibiotics. The presence of *B*. *fragilis* induced T helper 1 (Th1) responses and promoted dendritic cell (DC) maturation (IL-12 production) in tumor-draining lymph nodes and within the tumor itself, respectively. Interestingly, treatment with anti-CTLA-4 was also able to induce an immune response against these *Bacteroides* species, which raised the hypothesis of potential molecular mimicry between tumor antigens and specific commensal bacterial species. Similar results were obtained when feces from melanoma patients treated with ipilimumab were transplanted into germ-free mice, which resulted in the induction of a marked response to anti-CTLA-4 and decreased tumor growth.

Heterogeneous and transitory responses to immune checkpoint inhibitors are often observed in patients with melanoma^[64]^. Sivan *et al.*^[65]^ hypothesized that intestinal microbiota can be a possible modulator of the immune response in melanoma patients. In an animal model, tumor growth of subcutaneously injected B16.SIY cells was different in genetically similar C57BL/6 mice that harbored different commensal microbes. The difference in B16.SIY cell growth rate was due to different spontaneous antitumor immunity characterized by enhanced tumor-specific T cells responses and intratumoral CD8^+^ T cells infiltration. The authors found that *Bifidobacterium* application was associated with the antitumor effect, as it was able to activate DCs, possibly through soluble signaling factors disseminating systemically, which in turn elicited the antitumor effector functions of CD8^+^ T cells. Furthermore, oral administration of *Bifidobacterium* resulted to be effective in reducing tumor growth to the same extent as anti-PD-L1 treatment, and the combination of these two treatments almost completely inhibited tumor growth.

More recently, other studies focused on the role of the microbiota in regulating the efficacy and resistance of ICI therapy for cancer treatment. Routy *et al.*^[66]^ found that primary resistance to ICI therapy can be associated with altered gut microbiota composition, as antibiotic treatment inhibited the efficacy of ICI therapy in patients with advanced cancer. Patients with advanced non-small cell lung carcinoma, renal cell carcinoma, and urothelial carcinoma treated with a combination of broad-spectrum antibiotics (including β-lactamase-inhibitors, fluoro-quinolones, or macrolides) 2 months before, or one month after the first administration of anti-PD1/PD-L1 therapy, had a poorer clinical prognosis compared to patients who did not receive antibiotics. These antibiotics are generally prescribed for common indications including dental, urinary, and pulmonary infections, and can transiently alter the composition of gut microbiota. A metagenomic analysis of patients’ stools revealed a relative abundance of *Akkermansia muciniphila* was associated with improved clinical responses to ICI. As one of the most abundant bacteria in the ileum microbiota^[67]^, *A. muciniphila* can induce DCs to secrete IL-12 and increase recruitment of CD4^+^ T central memory cells expressing small intestine-associated chemokine receptor CCR9 and Th1-associated chemokine receptor CXCR3 to mice tumors.

Another work by Matson *et al.*^[68]^ showed that the composition of microbiota could contribute towards identifying cancer patients who may benefit from anti-PD-1-based immunotherapy. A metagenomic analysis of baseline stool samples from metastatic melanoma patients before immunotherapy revealed an association between the composition of commensal microbes and clinical responses. Specifically, *Bifidobacterium longum*, *Collinsella aerofaciens*, and *Enterococcus faecium* were more abundant in patients who responded to anti-PD-1 immunotherapy.

A similar study by Gopalakrishnan *et al.*^[69]^ compared the gut microbiome between responder and non-responder metastatic melanoma patients treated with anti-PD-1 immunotherapy. Significant differences in both the diversity and composition of the microbiome were found between the two groups of patients, with a relative abundance of bacteria of Clostridiales order and *Ruminococcaceae* family in the responder group. Furthermore, a higher density of CD8^+^ T cell infiltration was observed in tumor tissues of responder patients and this was correlated with the abundance of the *Faecalibacterium* genus (*Ruminococcaceae* family). Analysis of the systemic immune response showed a higher frequency of effector CD4^+^ and CD8^+^ T cells in circulation and a preserved ability of cytokine production in patients with a higher abundance of *Ruminococcaceae* in gut microbiota. On the other hand, non-responder patients had enrichment in *Bacteroides thetaiotaomicron*, *Escherichia coli*, and *Anaerotruncus colihominis*. Furthermore, non-responder patients with a higher abundance of *Bacteroidales* had increased levels of regulatory T (Treg) cells and myeloid-derived suppressor cells in the systemic circulation, with reduced cytokine production ability.

A systematic analysis identified human-associated microbial species that have the specific ability to modulate Treg cell responses. Faith *et. al*^[70]^ showed that, in *in vivo* study using mice monocolonized by specific bacterial strains, four members from the *Bacteroides* genus (*B*. *caccae*, *B*. *thetaiotaomicron*, *B*. *vulgatus*, and *B*. *massiliensis*), one from the *Parabacteroides* genus (*P*. *distasonis*), and *E*. *coli* from the *Escherichia* genus were able to significantly increase the proportion of Foxp3^+^ Treg cells among CD4^+^ T cells.

In summary, these results provide evidence to support the crucial role of the gut microbiota in regulating the effector functions of immune cells and thus, control of the efficacy of ICI therapy. The presence of specific microbial species is also essential for an effective response to, or failure of treatment.

## SOLUBLE SIGNALING FACTORS FROM GUT MICROBIOTA REGULATE IMMUNE CELLS

### Toll-like receptor activation

We have discussed above that soluble signaling factors released from commensal microbiota may regulate the activation of immune cells in the TME or tumor-draining lymph nodes, and thus the efficacy of immune therapy. These soluble signaling factors include microbe-associated molecular patterns (MAMPs) or pathogen-associated molecular patterns (PAMPs), which are conserved, specific molecules derived from microbes. They can bind to pattern recognition receptors (PRRs) such as Toll-like receptors, cytosolic NOD-like receptors, C-type lectin receptors, and RIG-I-like receptors. PRRs are expressed by immune cells of the innate (macrophages and DCs) and adaptive (T and B cells) immune system^[28,71,72]^. Therefore, microbiota-derived MAMPs or PAMPs, such as LPS^[73]^, peptidoglycan^[74]^, or flagellin^[75]^, can enter the circulation through the mucosal barrier to modulate immune responses to cancer. Nucleic acids, such as unmethylated CpG released by bacteria, can bind to TLR9 and control differential T cell responses by regulating Th1 and Th2 polarization^[76]^, the frequency of CD4^+^Foxp3^+^ Tregs, and IL-17-IFN-γ-producing effector T (Teff) cells^[77]^. Bacteroidetes are one of the major phyla of the commensal microbiota and they can control inflammation by regulating the differentiation of Tregs. Capsular polysaccharide A released by *Bacteroides fragilis* (*B. fragilis*) has immunoregulatory properties that mediate the conversion of CD4^+^ T cells into Foxp3^+^ Tregs through TLR2-mediated signaling. These cells have increased suppressive capacity through higher production of the anti-inflammatory cytokine IL-10^[78]^.

In summary, soluble signaling factors released from specific bacterial species of the gut, which include structural components of bacteria, such as MAMPs, can induce different immune responses with opposing effects on the efficacy of cancer therapy.

### Metabolite receptor activation

Other soluble signaling factors from gut microbiota can regulate cancer immune responses. Growing evidence highlight the importance of bacterial metabolites in the profound regulation of immune cells^[79]^ and accordingly, the efficacy of cancer immunotherapy. Immune cells express receptors specific for bacterial metabolites: purinergic receptors (P2X_7_) detect adenosine triphosphate (ATP) and nicotinamide adenine dinucleotide; GPR43 and GPR41 detect short-chain fatty acids (SCFAs); membrane bile acid receptor (M-BAR/TGR5) and farnesoid X receptor detect bile acid and xenobiotic metabolites; and aryl hydrocarbon receptor precursor and pregnane X receptor detect tryptophan, indole, bile acid and toxicant metabolites^[80]^. Gut bacteria produce SCFAs, such as butyrate, acetate, and propionate, through anaerobic fermentation of carbohydrates. SCFA receptors are expressed on macrophage, dendritic cells, and neutrhophils, which can regulate T and B cell-mediated responses. SCFAs also play an important role in the generation and modulation of Tregs^[81]^. Butyrate, produced during starch fermentation, induces extrathymic differentiation of Tregs and propionate potentiates the generation of Tregs in the periphery^[82]^. SCFAs can also regulate T cell differentiation into T helper 17 (Th17), Th1, and IL-10-producing T cells by their histone deacetylase inhibitor activity^[83]^. Indeed, butyrate can induce differentiation of Tregs both *in vitro* and *in vivo* by enhancing the acetylation of histone H3 in the promoter region and intragenic enhancer elements of the *FoxP3* locus termed conserved noncoding sequence^[84]^.

Roughly, one-third of patients treated with anti-CTLA-4 develop immune-mediated colitis induced by mucosal immune dysregulation^[85]^. *Bacteroides* can induce the expansion of Tregs^[78]^, which play an important role in regulating the development of colitis^[86]^. Increased representation of members of the Bacteroidetes phylum (Bacteroidaceae, Rikenellaceae and Barnesiellaceae families) inhibit the development of colitis induced by anti-CTLA-4 immunotherapy and this is associated with the biosynthesis of B vitamins^[87]^.

In summary, metabolites produced by bacteria have important effects on the regulation of immune cell functions and accordingly, the efficacy of cancer therapy.

## GUT MICROBIOTA REGULATES THE RESPONSE TO CHEMOTHERAPY

Besides regulating the response to immunotherapy, gut microbiota can also modulate the efficacy of chemotherapy. As discussed above, antibiotic treatment can reduce the efficacy of CpG-oligodeoxynucleotides and anti-IL-10R treatment in retarding tumor growth and prolonging survival of mice^[62]^. Besides regulating the response to immunotherapy, the authors have shown that the absence of commensal bacteria, either by treatment with an antibiotic cocktail or rearing mice in germ-free conditions, resulted in an impaired response to treatment with oxaliplatin, a cytotoxic cancer chemotherapy that forms platinum DNA adducts and intrastrand cross-links^[88,89]^. The absence of gut microbiota was also associated with the reduction of the expression of *Cybb* gene, which encodes for NADPH oxidase 2 (Nox2) that in turn determined a decreased generation of reactive oxygen species (ROS) by tumor-associated myeloid cells (neutrophils and macrophages) and ROS-mediated oxaliplatin genotoxicity. The underlying mechanism involved could be associated with microbial products sensed by myeloid cells in a TLR4-independent fashion.

Cyclophosphamide (CTX) is an alkylating chemotherapy drug that interferes with DNA replication by forming intrastrand and interstrand DNA crosslinks^[90]^. Besides controlling tumor cell apoptosis^[91,92]^, the therapeutic efficacy of CTX is also due to its ability to induce immunogenic cancer cell death, leading to stimulation of antitumor immune responses. Viaud *et al.*^[93]^ showed that CTX treatment of mice bearing subcutaneous cancers (B16F10 melanoma cells and MCA205 sarcoma cells) induced increased permeability of the intestinal mucosa with consequent translocation of distinct commensal bacteria (gram-positive *Lactobacillus johnsonii*, *Lactobacillus murinus* and *Enterococcus hirae*) into mesenteric lymph nodes and the spleen. Gram-positive bacteria could induce polarization of CD4^+^ T cells towards IFN-γ-producing Th1 and “pathogenic” Th17 (pTh17, expressing both IFN-γ and IL-17) cells, which are important effector cells in controlling cancer growth^[94]^. Importantly, both broad-spectrum (ATB) and gram-positive specific antibiotics (e.g., vancomycin) considerably reduced the anticancer efficacy of CTX against MCA205 sarcoma and P815 mastocytoma. Similar results were obtained using a transgenic lung cancer mouse model, where a reduction of tumor-infiltrating CD3^+^ T and Th1 cells was seen.

A study by Daillère *et al.*^[95]^ revealed that two different commensal bacteria located in different parts of the intestine could ameliorate the efficacy of CTX. In tumor-bearing mice treated with CTX, the gram-negative *Barnesiella intestinihominis*, residing in the colon, induced systemic CD4^+^ Th1 cells and type 1 CD8^+^ T (Tc1) cells and increased the proportion of intratumoral IFN-γ-producing γδT cells. In contrast, the gram-positive *E. hirae*, which resides in the small intestine, induced systemic pTh17 cells and increased the intratumoral CD8^+^ cytotoxic T lymphocytes (CTL): Tregs ratio. These effects were subsequently translated into effective anticancer responses to CTX. Furthermore, the presence of memory CD4^+^ CD45RO^+^ Th1 cells against *E. hirae* and *B. intestinihominis* was predictive of a longer progression-free survival in lung cancer patients after platinum-based chemotherapy and, in the treatment of ovarian cancer patients resistant to platinum-based chemotherapy with CTX.

More recently, the intratumor presence of bacteria was found to be responsible for chemotherapeutic drug resistance. Geller *et al.*^[96]^ found that the presence of *Mycoplasma hyorhinis* (*M*. *hyorhinis*) in human dermal fibroblasts determined resistance to gemcitabine both *in vitro* and *in vivo* and this was due to the metabolization of gemcitabine into its deaminated inactive metabolite 2’,2’-difluorodeoxyuridine. However, *Mycoplasma sp.* was not the only bacteria capable of inducing resistance to gemcitabine. Indeed, the author revealed that bacteria belonging to the Gammaproteobacteria class expressed the long form bacterial enzyme cytidine deaminase (CDD_L_), which mediates gemcitabine deamination and its inactivation. In an animal model of colon cancer, the intratumor presence of CDD_L_-expressing bacteria conferred resistance to gemcitabine and the elimination of bacteria by ciprofloxacin treatment restored the response to the chemotherapeutic drug. Furthermore, analysis of a cohort of 113 pancreatic ductal adenocarcinoma (PDAC) tissues revealed the presence of Gammaproteobacteria (most of them belonging to *Enterobacteriaceae* and *Pseudomonadaceae* families) in 86 patients’ samples (76%), possibly due to retrograde migration from the duodenum to the pancreas. Bacteria from fresh PDAC could also render both colon cancer cell lines RKO and HCT116 fully resistant to gemcitabine, in an *in vitro* co-culture model.

In summary, gut microbiota can regulate the response to chemotherapy either by affecting the effector mechanism of the drug (e.g., ROS production), modulating the induction of its immunogenic properties, or metabolizing it into an inactive form.

## GUT MICROBIOTA REGULATES THE EXPRESSION OF HOST CELLS MIRNAS THAT MODULATE THE RESPONSE TO CANCER THERAPY

Our discussion thus far has suggested that gut microbiota can regulate the response to cancer therapy by modulating the functions of immune cells against cancer cells, or affecting the efficacy of chemotherapeutics drugs. Growing evidence reveals that gut bacteria play a role in the modulation of gene expression in host cells by regulating their miRNA expression^[97,98]^. Dalmasso *et al.*^[99]^ showed that one miRNA in the ileum (miR-298) and eight miRNAs in the colon (upregulated: miR-128, miR-200c, miR-342–5p; downregulated: miR-465c-5p, miR-466d-3p, miR-466d-5p, miR-665 and miR-683) were differentially expressed in the microbiota of colonized mice compared with germ-free mice. Importantly, the microbiotamediated downregulation of miR-665 induced the upregulation of its target gene, the ATP-binding cassette transporter Abcc3, both at mRNA and protein levels in the colon. Notably, the overexpression of Abcc3 in colon cancer can contribute to the acquisition of drug resistance^[100]^.

Commensal bacteria can also regulate miRNA expression in dendritic cells and accordingly their functions. For instance, the engagement of TLR1/2, TLR4, TLR5, TLR9 and NOD2 induces the downregulation of miR-10a through the MyD88-dependent pathway^[101]^. MiR-10a also targets the *IL-12/IL-23p40* gene, which is a key regulator of the induction of Th1 cell-mediated immune responses and increasd cytotoxic activity of CD8^+^ T and natural killer cells. These immune cell-mediated functions are crucial for effective anticancer immune responses.

Recently, an over-representation of *Fusobacterium nucleatum* (*F*. *nucleatum*) was found in the tissue of colorectal cancer (CRC) patients compared with matched normal tissue^[102]^. Interestingly, Yu *et al.*^[103]^ found a higher abundance of *F*. *nucleatum* in the tissue of CRC patients with recurrent disease compared with non-recurrent patients after chemotherapy. Furthermore, high levels of *F*. *nucleatum* were associated with shorter recurrence-free survival. To investigate the potential mechanisms behind *F*. *nucleatum*-induced chemoresistance, the authors compared miRNA expression profiles between CRC tissues with a high amount of *F*. *nucleatum* from recurrent patients and CRC tissues with a low amount of *F*. *nucleatum* from non-recurrent patients. Among the 68 miRNAs that were differentially expressed, two specific miRNAs showed the most significant downregulation in CRC tissues with a high amount of *F*. *nucleatum*: miR-4802 and miR-18a*. These two miRNAs target ULK1 and ATG7, both of which are mediators of autophagy. The *F*. *nucleatum*-mediated downregulation of miR-4802 and miR-18a* was associated with reduced apoptosis induced by oxaliplatin and 5-FU through the activation of autophagy both *in vitro* and *in vivo*. The acquisition of chemoresistance mediated by *F*. *nucleatum* was dependent on the activation of TLR4/MYD88 signaling.

In summary, these studies provide insight into the role of soluble signaling factors released by gut microbiota on the modulation of drug resistance through the regulation of miRNA expression in host cells.

## EVS MEDIATED CROSS TALK COMMUNICATION BETWEEN BACTERIA AND HOST CELLS

Due to the high number of molecules with regulatory ability, EVs play an important role in cell-to-cell communication and the regulation of biological function in both normal and cancer cells^[29]^. Bacteria are able to generate EVs carrying molecules with signaling properties. The presence of nucleic acids inside bacterial EVs and the ability to mediate intercellular genetic transfer was first detected in 1989^[104]^. The mechanism of biogenesis of EVs by bacteria remains poorly understood and less well-known compared to that of eukaryotic cells. Bacterial EVs can be generated by both gram-positive^[105–107]^ and gram-negative bacteria^[108]^. Most of our understanding of the biogenesis of bacterial EVs originate from studies on gram-negative bacteria^[108]^, which generate EVs from the outer membrane and accordingly, are named outer membrane vesicles (OMVs). These may range from 20 to 250 nm in diameter and contain components derived from the outer membrane (OM), inner membrane, and periplasmic space, which includes proteins, lipoproteins, phospholipids and LPS. OMVs are generated by blebbing outwards from the OM, during which process they include soluble components inside and adherent material, on the external surface^[109]^. EVs produced by gram-positive bacteria are structurally similar to OMVs, with sizes ranging from 10 to 400 nm^[107]^. They carry bacterial components including nucleic acid, proteins, lipids, enzymes and toxins. Compared with the well-elucidated mechanism of generating OMVs, the secretion of gram-positive EVs is still disputed, with an evidence-supported hypothesis that describes the enzyme-mediated degradation of the bacterial wall to facilitate EV release^[105,106]^. The loading of cargo inside gram-positive EVs and OMVs may represent an active metabolic process^[110]^, as marked differences in RNA content were measured between bacterial EVs and their producer cells^[43,111,112]^.

Bacterial EVs are enriched in sRNAs and msRNA^[41,51,52]^ that have regulatory functions similar to miRNA in eukaryotic cells. Therefore, EV-mediated delivery of these RNA molecules can potentially regulate specific genes in recipient human cells^[54]^. A recent study reported that OMVs from *Pseudomonas aeruginosa* can mediate the delivery of a specific sRNAs (sRNA52320) to human bronchial epithelial cells and target two mitogen-activated protein (MAP) kinases (MAP3K7 and MAP2K4) to regulate the LPS-stimulated mitogen-activated protein kinase (MAPK) signaling pathway^[51]^. Another study showed that the periodontal pathogens *Aggregatibacter actinomycetemcomitans*, *Porphyromonas gingivalis*, and *Treponema denticola* express msRNAs that can be delivered to fibroblast cells through OMVs. Furthermore, transfection of Jurkat cells with synthetic msRNAs reduced the expression of IL-5, IL-13 and IL-15^[52]^. These studies confirm that bacterial EVs have the ability to deliver short RNA molecules with regulatory functions to human cells and modulate gene expression. This microbial biological function may support the hypothesis that bacterial EVs might potentially modulate the expression of genes regulating resistance to chemotherapy and immunotherapy.

Interestingly, host cells can also participate in inter-kingdom communication through the delivery of miRNAs encapsulated within human cell-derived EVs to bacteria [Figure 1]. Host intestinal epithelial cells secrete EV-encapsulated miRNAs that can be detected in the feces of mouse (the most abundant: miR-1224, miR-2146, miR-2134, miR-483, miR-710, miR-2141, miR-720, miR-155 and miR-34c) and human (the most abundant: miR-1246, miR-601, miR-630, miR-2116–5p, miR-320e, miR-1224–5p, miR-155–5p and miR-1945p). EV-encapsulated miRNAs can then be taken up by *F. nucleatus* and *E. coli* and alter their expression of genes controlling their growth, with the result of maintaining the normal physiological balance of gut microbiota. In particular, hsa-miR-515–5p promotes the growth of *F. nucleatus* while hsa-miR-1226–5p promotes the growth of *E. coli*^[113]^.

Collectively, these studies provide supporting evidence that a potential two-way EV-mediated communication may exist between bacteria and human host cells, resulting in reciprocal regulation of gene expression and accordingly, biological cell function. An example of such reciprocal regulation between gut bacteria and host cells with an effect on the development of drug resistance is illustrated by *F. nucleatus*. High amounts of *F. nucleatus* in the gut of CRC patients induce the activation of autophagy in CRC cells through the TLR4/MYD88-mediated downregulation of miR-18a* and miR-4802, which in turn promotes chemoresistance of CRC cells to chemotherapeutic drugs (oxaliplatin and 5-FU)^[103]^. On the other hand, intestinal epithelial cells can control the growth of *F. nucleatus* through the delivery of miRNAs, such as hsa-miR-515–5p^[113]^. In CRC patients, this equilibrium can be potentially compromised by the altered expression of miR-515–5p, that might affect *F. nucleatus* proliferation and consequent response to chemotherapeutic drugs, and thus prognosis^[114]^.

## CONCLUSIONS

The development of drug resistance represents the most important cause of cancer treatment failure and is responsible for the poor prognosis of cancer patients. Growing evidence demonstrates that tumors are characterized by a high degree of molecular heterogeneity^[115]^ and cancer treatment can promote therapyinduced selection for a previously minor subset of resistant cells^[116]^. Different mechanisms are responsible for drug resistance. Some arise from intrinsic modification of cancer cells causing the alteration of genes that control specific pathways involved in treatment resistance such as drug transport and metabolism, alteration of drug targets, DNA damage repair, deregulation of apoptosis, autophagy, and prosurvival signaling^[117]^. Other mechanisms are included in the complex network of signaling the TME^[118]^.

Recently, the gut microbiota has received increasing attention for its role in regulating the efficacy of both immunotherapy and chemotherapy. Indeed, since the first evidence suggestive of the role of microbes in cancer treatment in 1890^[59]^, recent work has started to show that gut microbiota can have a remarkable effect on cancer therapies. After the advent of ICI therapy and promising evidence of its curative potential when used in combination with chemotherapy^[119]^, it has been shown that specific species of the gut microbiota can regulate the efficacy of ICI responses. Specific *Bacteroides* species (*B*. *thetaiotaomicron* and *B*. *fragilis*) are essential for an effective response to anti-CTLA-4^[63]^. The presence of *Bifidobacterium* in combination with anti-PD-L1 treatment can result in almost complete inhibition of melanoma tumor growth^[65]^. The presence of *A. muciniphila*, one of the most abundant bacteria in the ileum microbiota, is associated with clinical response to anti-PD-1/PD-L1 therapy^[66]^. Similarly, patients who respond to anti-PD-1 therapy have high levels of *Bifidobacterium longum*, *Collinsella aerofaciens*, and *Enterococcus faecium*^[68]^. Patients with melanoma who respond to anti-PD-1 therapy have a relative abundance of bacteria of the *Ruminococcaceae* family, whereas patients who do not respond are enriched in *Bacteroides thetaiotaomicron*, *Escherichia coli*, and *Anaerotruncus colihominis*^[69]^. As reported in the studies above, it is important to note that the use of antibiotics induces dysbiosis or elimination of gut microbiota and accordingly, impairs the efficacy of ICI therapy. Cancer patients are already at higher risk of bacterial infection and sepsis during treatment. Indeed, chemotherapeutic drugs^[120]^ and ICI therapy^[121,122]^ often cause intestinal mucositis and treatment-induced intestinal barrier dysfunction due to injury of the mucosal barrier with consequent translocation of bacteria across the gut into the systemic environment [Figure 1]. Furthermore, neutropenia is often associated with cancer treatment^[120,123]^. These two complications require prophylactic antibiotic therapy to prevent sepsis during chemotherapy^[124,125]^. Because the alteration of the physiological equilibrium of gut microbiota induced by prophylactic antibiotic therapy can affect the efficacy of chemotherapy and immunotherapy, a careful approach should be considered with regard to prophylactic antibiotics for cancer patients. Furthermore, intestinal barrier dysfunction is associated with increased levels of systemic LPS-positive bacterial EVs that induce the secretion of pro-inflammatory mediators (IL-6, IL-8, MCP-1 and MIP-1α) by peripheral blood mononuclear cells^[126]^ with consequent increased risk of sepsis or toxic shock syndrome^[127]^. Therefore, the use of selective antibiotic therapy to target specific harmful bacterial species and spare those associated with the effectiveness of cancer therapy along with a concomitant administration of probiotics, prebiotics to rebalance the gut microbiota should be taken in consideration^[128]^.

It is noteworthy to observe that specific bacterial species are associated with effective responses to cancer therapy. Thus, it could be hypothesized that besides common MAMPs or PAMPs that are conserved molecules derived from bacteria, other specific bacteria-derived components may be involved in regulating the efficacy of cancer treatment. That bacteria-specific protein antigens are able to elicit and sustain a cancer immune response could be a possible explanation. Additionally, bacteria-specific nucleic acid sequences that are able to activate TLR3–8-9-mediated immune responses could also be involved.

An additional level of complexity is added by bacterial EVs, which can carry bacterial components and have the ability to regulate gene expression in host recipient cells. Potentially, shuttling of sRNAs through bacterial EVs to host cells could represent an additional mechanism to either inhibit or enhance the response to chemotherapy and immunotherapy. Thus, EV-mediated delivery of bacterial sRNAs to cancer cells has the potential to represent a new field of research for the study of response to cancer therapies. Furthermore, due to the two-ways nature of EV-mediated inter-kingdom communication, either normal or host cancer cells may potentially affect the microbiota composition and in turn, regulate the efficacy of cancer therapies.

In conclusion, the gut microbiota represents a new player in the regulation of cancer therapy efficacy. EV-mediated inter-kingdom communication could also represent a new research field to better understand the relationship between commensal bacteria and the human host to improve the efficacy and reduce toxicity of cancer therapies.

## Figures and Tables

**Table 1.** Roles of bacteria in regulating the response to cancer therapies

Bacterial species	Cancer type	Therapy	Mechanism	Ref.
*Streptococci*	Different types	Tumor cell killing	Unknown	[59]
*Mycobacterium bovis*	Human bladder	Reduction of tumor progression	Unknown	[60]
*Lactobacillus casei*	Human bladder	Decrease of recurrence	Unknown	[61]
*B. thetaiotaomicron and B. fragilis*	Mouse sarcoma, melanoma, colon	Response to anti-CTLA-4	Th1 induction, DC maturation (IL-12 production)	[63]
*Bifidobacterium*	Mouse melanoma	Response to anti-PD-L1	DC activation, stimulation of CD8^+^ T cell effector functions	[65]
*A. muciniphila*	Human NSCLC, RCC, UC	Response to anti-PD-1/PD-L1	DC activation, increased CD4^+^ TCM tumor infiltration	[66]
*Bifidobacterium longum, Collinsella aerofaciens, and Enterococcus faecium*	Human melanoma	Response to anti-PD-1	Cancer immune response	[68]
*Faecalibacterium, Ruminococcaceae*	Human melanoma	Response to anti-PD-1	Increased CD8^+^ T cell tumor infiltration	[69]
*Bacteroides thetaiotaomicron, Escherichia coli, Anaerotruncus colihominis*	Human melanoma	Response to anti-PD-1	Increased levels of Treg cells and MDSCs	[69]
*B. caccae, B. thetaiotaomicron, B. vulgatus, B. massiliensis, P. distasonis, E. coli*	Normal mouse colon	No therapy	Increase the proportion of Foxp3^+^ Treg cells among the CD4^+^ T cells	[70]
*Commensal microbiota*	Mouse lymphoma	Oxaliplatin	ROS-mediated cytotoxicity	[62]
*Lactobacillus johnsonii, Lactobacillus murinus, Enterococcus hirae*	Mouse melanoma, sarcoma	Cyclophosphamide	Induction of Th1, Th17-IFN-_γ_^+^, CD3^+^ T cell tumor infiltration	[93]
*E. hirae, B. intestinihominis*	Mouse sarcoma	Cyclophosphamide	Induction of Th1, CD8^+^ T cell tumor infiltration	[95]
Gammaproteobacteria	Mouse colon cancer	Gemcitabine	Intratumor gemcitabine deamination	[96]

NSCLC: non-small cell lung carcinoma; RCC: renal cell carcinoma; UC: urothelial carcinoma; TCM: T central memory; MDSCs: myeloid-derived suppressor cells; ROS: reactive oxygen species; DC: dendritic cell
